# AN OPEN PILOT STUDY OF A COMMUNITY-ENGAGED BRAIN HEALTH EDUCATION PROGRAM FOR CHINESE AMERICANS AGED 50 OR ABOVE

**DOI:** 10.1093/geroni/igae098.4118

**Published:** 2024-12-31

**Authors:** Kaipeng Wang, Carson De Fries, Xiaoyouxiang Li, Fei Sun, Jie Zhu

**Affiliations:** University of Denver, Denver, Colorado, United States; University of Denver, Denver, Colorado, United States; University of Denver, Denver, Colorado, United States; Michigan State University, East Lansing, Michigan, United States; Texas State University, San Marcos, Texas, United States

## Abstract

Chinese Americans, the largest Asian American subgroup in the U.S., face linguistic, cultural, and socio-economic barriers to dementia prevention. To promote brain health in this population, a community-engaged approach is essential. This study evaluates a community-engaged education program for Chinese Americans aged 50 or older, designed to enhance brain health knowledge and motivate lifestyle changes to prevent dementia. The program is structured into four phases. First, we assessed participants’ interests in brain health topics, availability, and preferred modes of delivery. Next, experts in the identified topics developed educational content and outcome assessments. The third phase involved implementing a six-session program over three months, offered in Mandarin both virtually and in-person. The sessions covered general knowledge about Alzheimer’s disease and related dementias, diet, sleep, physical exercise, health checks, and mindfulness. Finally, we evaluated the program’s feasibility and effectiveness using mixed methods, including pre-post surveys, session feedback questionnaires, and focus groups. Seventy-seven participants completed the pretest survey, and 47 (61%) attended at least four sessions and completed the post-test survey. The results of paired t-tests showed statistically significant improvements in knowledge of brain health, diet, exercise, and health checks, as well as increased motivation for lifestyle changes. While supporting these results, qualitative findings further revealed the applicability, understandability, comprehensiveness, cultural relevance, elder friendliness of the sessions, and participants’ motivation for attending and self-improvement. Findings highlight feasibility and effectiveness of a community-engaged education approach in brain health promotion and lifestyle change and underscore the need for continued community outreach among Chinese Americans.

